# Development of an ELISA Using Recombinant Chimeric SM Protein for Serological Detection of SARS-CoV-2 Antibodies

**DOI:** 10.3390/mps9010004

**Published:** 2025-12-22

**Authors:** Gulnur Nakhanova, Olga Chervyakova, Kamshat Shorayeva, Aisha Issabek, Sabina Moldagulova, Asankadyr Zhunushov, Aknur Ulankyzy, Aigerim Zhakypbek, Alisher Omurtay, Aziz Nakhanov, Zharkinay Absatova, Yeraly Shayakhmetov, Kuanysh Jekebekov, Temirlan Baiseit, Aslan Kerimbayev

**Affiliations:** 1 Research Institute for Biological Safety Problems LLP, National Holding QazBioPharm JSC, Almaty 080409, Kazakhstan; gal.gulnur@mail.ru (G.N.); ovch@mail.ru (O.C.); a.issabek@biosafety.kz (A.I.); sabina_moldagulova@mail.ru (S.M.); a.ulankyzy@biosafety.kz (A.U.); aigerim.zhakypbek@mail.ru (A.Z.); a.omurtay@biosafety.kz (A.O.); a.nakhanov@biosafety.kz (A.N.); zh.absatova@biosafety.kz (Z.A.); e.shayahmetov@biosafety.kz (Y.S.); k.zhekebekov@biosafety.kz (K.J.); t.baiseit@biosafety.kz (T.B.); a.kerimbayev@biosafety.kz (A.K.); 2 Institute of Biotechnology, National Academy of Sciences of the Kyrgyz Republic, Bishkek 720071, Kyrgyzstan; junushov@mail.ru

**Keywords:** recombinant chimeric protein, cloning, expression, purification, enzyme-linked immunosorbent assay

## Abstract

The emergence and spread of coronavirus infections have created a necessity to develop serological methods for assessing population immunity. The enzyme-linked immunosorbent assay (ELISA) remains one of the most accessible and informative approaches for these purposes. The choice of recombinant proteins plays an important role in the sensitivity and specificity of the test system, and in this regard, the creation of a domestic ELISA based on the chimeric SM protein to the SARS-CoV-2 virus is relevant. In this work, a recombinant chimeric SM protein expressed in the *E. coli* system and purified using metal-affinity chromatography on Ni-NTA agarose was constructed and presented for the first time. An ELISA test system was developed and tested using panels of positive and negative sera, including samples obtained before the COVID-19 pandemic. The obtained sensitivity (90.48%) and specificity (93.65%) indicators with a ROC curve AUC = 0.9623 (OD450 = 0.213) indicate the diagnostic accuracy of the test system. The positive diagnostic ratio (LR+) = 14.25.0 indicates the reliability of a positive result. The domestically developed ELISA test system can be used for serological monitoring and assessment of the immune status of the population.

## 1. Introduction

The epidemiological situation associated with COVID-19 continues to pose a serious threat to public health worldwide [1]. In the context of the spread of the SARS-CoV-2 virus, antibodies play a key role in diagnosis and in assessing the immune response after infection or vaccination. The presence of antibodies for the SARS-CoV-2 virus is considered one of the most important indicators of the immune protection of the population during a pandemic, contributing to the effective control of the spread of infection and reducing its consequences [2,3,4,5]. Antibodies not only help neutralise the virus but also allow tracking the level of immune activity in people who have recovered from the disease and those who have been vaccinated. A key aspect of this strategy is the reliability of diagnostic methods.

Despite the widespread use of serological tests, there is a need to improve the sensitivity and specificity of these test systems. Improving the quality of serological diagnostics is crucial for accurately identifying infected individuals and assessing the population immune response [6,7,8]. In addition, the SARS-CoV-2 virus is known to be prone to rapid mutations, leading to genetic variability in circulating virus strains. In turn, these mutations have affected the diagnostic accuracy of serological tests [9]. Therefore, an approach based on the use of chimeric or combined proteins capable of increasing the diagnostic effectiveness of ELISA is becoming relevant.

Severe acute respiratory syndrome coronavirus (SARS-CoV-2) is an enveloped single-stranded positive-sense RNA virus of the genus Betacoronavirus. The viral genome encodes four major structural proteins: spike (S), envelope (E), membrane (M), and nucleocapsid (N). The S protein, which forms a crown-like structure typical of all coronaviruses, is a helical glycoprotein belonging to the class of type I viral fusion proteins, which aggregate into a homotrimer from monomers with a mass of ~180 kDa. The SARS-CoV-2 M protein consists of 222 amino acids (aa) and is postulated to span the viral membrane three times and have a short, single-glycosylated N-terminal ectodomain and a long C-terminal endodomain. The coronavirus membrane protein (M) plays a central role in virion morphogenesis. The M protein organises the components of the viral membrane, and the interactions of the M protein with itself and with the N protein control the assembly and budding of the virus.

In our study, the choice of the S protein was due to the high sensitivity of the diagnostic test when used, despite mutations in this protein. Studies show that some regions of the S protein, such as the S2 domain, remain conserved [10].

The choice of M protein was based on its conservative N-terminal transmembrane region protruding above the membrane. Early studies with SARS-CoV suggest that neutralising antibodies specific to the S protein, as well as binding antibodies to the M protein, may recognise conformational epitopes that are involved in the binding of virions to cell receptors [11]. Studies conducted to find suitable peptides for recognising the severity of COVID-19 have shown the presence of an epitope on the M protein for which a strong humoral response directly correlated with disease severity [12,13]. One of the limitations of this study, according to the authors, was that they only studied short peptides, whereas longer peptides may be more informative. Therefore, in this study, we used a chimeric SM protein of the SARS-CoV-2 virus, combining epitopes of the S (Spike) and M (Membrane) proteins. The S protein has high immunogenic activity and is the main target of neutralising antibodies, making it a central component of virtually all existing COVID-19 vaccines. The M protein is more conservative among different SARS-CoV-2 strains and, as shown, elicits a significant antibody response in patients: antibodies to linear epitopes of the M protein are detected in both the acute and recovery phases of the disease [14].

The use of chimeric or combined antigens in diagnostic tests allows for high sensitivity and specificity. It has been shown that the use of a chimeric protein containing the conserved regions of the S1 and N proteins achieved a better combination of sensitivity and specificity compared to the use of S1 or N proteins alone [15]. In addition, the inclusion of various antigens in the development of ELISA allows for the diversity of immune responses to be taken into account and reduces the likelihood of cross-reactivity with antibodies to other viruses or non-specific serum components. This approach improves test accuracy by combining the detection of even weak antibodies to the SARS-CoV-2 virus with the minimisation of false positive signals. [16,17]. Chimeric proteins based on 3 or 4 proteins demonstrated high reactivity and had high specificity and sensitivity [18]. This can be very relevant for mass screening, epidemiological monitoring, and in cases where it is necessary to ensure the most complete detection of antibodies in various clinical and post-vaccination samples.

Thus, the aim of this work is to create and evaluate the effectiveness of an ELISA test system based on the chimeric SARS-CoV-2 SM protein we have developed, as well as to determine its sensitivity, specificity, and effectiveness in detecting antibodies to SARS-CoV-2. Combining the epitopes of S and M proteins in a single chimeric SM protein allows the broad neutralising activity of the S protein to be combined with the conservative and stable immunogenic properties of the M protein, potentially providing more universal detection of antibodies. These particular proteins were chosen because of their key role in immune defence and their potential ability to cover a wide range of SARS-CoV-2 strains.

## 2. Materials and Methods

Virus: The SARS-CoV-2/KZ Almaty/04.2020 strain of the SARS-CoV-2 virus, the causative agent of COVID-19 infection, was isolated in April 2020 from a patient in Almaty, Republic of Kazakhstan. The SARS-CoV-2 virus strain (GenBank ID: MZ379258.1), deposited in GenBank and obtained from the Microorganism Collection of the Research Institute for Biological Safety Issues, was cultured in the Vero cell line for further studies, including virus neutralisation testing [19,20].

Bacteria: *E. coli* TOP10 was used to obtain and propagate recombinant plasmid DNA; strain ER2566 (Invitrogen, Walthan, MA, USA) was used to express the target protein.

Construction of a plasmid for recombinant protein expression: The study used plasmid vectors pGEM-T and pET32b(+). Nucleotide sequence synthesis was performed using a two-round PCR method [21]. Oligonucleotides were designed using Vector NTI 10.0.1 software. Nucleotide sequences were sequenced using the v3.1 Cycle Sequencing Kit BigDye Terminator on a Genetic Analyser 3130xl DNA sequencer, Applied Biosystems, Hitachi. The sequences were analysed using Vector NTI 10.0.1 software.

Recombinant protein expression. To produce recombinant protein, LB-amp100 medium was inoculated with an overnight *E. coli* ER2566 bacterial culture. The culture was incubated on a shaker (220–250 rpm) at 37 °C for 4 h until OD600 reached 0.8–1. Expression was induced by adding IPTG to a final concentration of 0.5 mM and incubation was continued under the same conditions for another 4 h. Cells were harvested by centrifugation at 4000 rpm.

Cell lysis. The cell pellet was resuspended in buffer 1 [10 mM Tris HCl pH 7.5, 1% Triton X-100, 150 mM NaCl] with the addition of 1% DOC at a rate of 10 mL of buffer 1 per 1 g of cells. Lysozyme was added to the resulting suspension to a final concentration of 1 mg/mL, along with 5 μL BenzNuclease to degrade nucleic acids, and the mixture was incubated for 1 h at 37 °C. Next, a freeze–thaw cycle was performed. It was centrifuged at 16,000× *g* at 4 °C for 20 min. The precipitate was washed once with the initial volume of buffer 1, and the supernatants were used in the next purification step. 

Purification of recombinant protein. Metal affinity chromatography was used to purify the recombinant protein. Ni-NTA agarose (Qiagen GmbH, Hilden, Germany) was used for metal affinity chromatography, which was performed on a BioLab 100 chromatography system (Hanbon, Huaian, Jiangsu, China). Recombinant protein sorption was carried out at a flow rate of 0.5 mL/min. The resin was then washed with buffer 2 [100 mM Tris HCl pH 7.5, 300 mM NaCl, 10 mM imidazole] at a flow rate of 2 mL/min. The protein was eluted with buffer 3 [100 mM Tris HCl pH 7.5, 300 mM NaCl, 100 mM imidazole] at a flow rate of 1 mL/min.

Sephadex G-25 Medium (Cytiva, Uppsala, Sweden) was used for buffer exchange. Protein yield was monitored using a flow-through UV detector. The purified concentrated protein was sterilised by filtration through membrane filters with a pore diameter of 0.22 μm. The concentration of purified recombinant protein was determined by the Lowry method [22], using bovine serum albumin (BSA) (Sigma, St. Louis, MO, USA) as a standard.

Sodium dodecyl sulphate-polyacrylamide gel electrophoresis (SDS-PAGE). Protein samples were separated by electrophoresis in 12% SDS-PAGE according to Laemmli [23] and visualised by staining with Coomassie G-250.

Western blot. For immunodetection, the recombinant protein was separated on a 12% SDS–polyacrylamide gel (SDS-PAGE), followed by electrotransfer to a nitrocellulose membrane (Invitrogen, Carlsbad, CA, USA). The membrane was then incubated for 2 h at room temperature with gentle agitation in blocking buffer (1× PBS containing 1% bovine serum albumin). The membrane was incubated with specific Anti-SARS-CoV-2 spike glycoprotein antibody (ab272504 Abcam) at 4 °C overnight, after which it was washed three times with TBS-Tween 20 solution (1× TBS supplemented with 0.1% Tween 20). Protein A, Biotin Conjugate (cat. number 203,195 Sigma-Aldrich, St. Louis, MO, USA) was used at a 1:2000 dilution with TBS-Tween 20 for 1 h at room temperature, then Avidin-Alkaline Phosphatase (cat. number A7294 Sigma-Aldrich, St. Louis, MO, USA) at a 1:50,000 dilution. After three washes with TBS-Tween 20, the membrane was developed using the BCIP/NBT phosphatase substrate system 1-Step NBT/BCIP (nitro blue tetrazolium/5-bromo-4-chloro-3-indolyl phosphate) (cat. number 34042, Thermo Fisher Scientific, Rockford, IL, USA) according to the manufacturer’s instructions.

Sampling. To optimise the enzyme-linked immunosorbent assay (ELISA) for antibodies to SARS-CoV-2, archived blood serum samples obtained during the COVID-19 pandemic and stored frozen in a biobank were used. The study included 126 serum samples, of which 63 were positive and 63 were negative. To confirm the status of the samples, both positive and negative sera were tested using a virus neutralisation test.

After collecting venous blood, serum was separated by centrifugation at 1500× *g* for 10 min, then aliquoted in five replicates of 300 μL to minimise repeated freeze/thaw cycles. Each aliquot was labelled with a unique code corresponding to the sample identification number. Storage was carried out at −80 °C in a biobank until analysis.

Sera obtained prior to the COVID-19 pandemic were used as negative samples, including from donors with confirmed antibodies to influenza viruses, seasonal HCoV (Human Coronavirus), parainfluenza, adenoviruses, and herpesviruses (Table 1). The sera were obtained from adult patients (aged 25–60 years), with an equal proportion of men and women. These samples had previously been tested for antibodies using commercial ELISA test systems and stored in the institution’s database, which allowed them to be used as reliable negative samples to assess the specificity of the test system and analyse possible cross-reactivity.

Enzyme immunoassay. Recombinant chimeric SM protein was used for ELISA, and the coating concentration of purified antigen was determined in the range from 1:20 (20 μg/mL) to 1:20480 (0.019 μg/mL) on Polysorp (Nunc, Thermo Fisher Scientific Nunc A/S, Kamstrupvej, P.O. Box 280 DK 4000 Roskilde, Denmark) plates. A 0.01 M carbonate-bicarbonate buffer (CBB), pH 9.6, was used as the protein dilution solution for coating. A 1% BSA in PBST [137 mM NaCl; 2.7 mM KCl; 10 mM Na2HPO4; 2 mM KH2PO4; 0.1% Tween-20 pH 7.5] was used as a blocking agent. The sera were diluted from 1:10 to 1:640 in PBST and incubated for 1 h at 37 °C, after which the wells were washed with PBST. The working concentration of the conjugate was determined by the signal-to-background ratio (S/B) and the difference in optical densities (ΔOD). For antibody detection, Pierce™ Recombinant Protein G, Peroxidase Conjugated (cat. no. 31499, Thermo Fisher Scientific, Rockford, IL, USA), was used at a 1:15,000 dilution and incubated for 30 min at 37 °C. After incubation with each component, the wells of the plate were washed four times, then 100 μL of 3,3’,5,5’-tetramethylbenzidine (TMB) substrate solution was added. The plates were incubated in a dark place at room temperature for 15 min. The reaction was stopped with 100 μL of 1 M H_2_SO_4_. The optical density (OD) was measured using an ImmunoChem-2100 microplate reader (High Technology Inc, Walpole, MA, USA) at a wavelength of 450 nm. The data were analysed considering the serial dilutions of both the antigen and the serum.

Virusneutralization Test (VNT). The presence and titers of neutralising antibodies were determined using a monolayer culture of Vero cells prepared in 96-well plastic plates. It was carried out using blood sera collected from humans, and the virus titre and blood serum titre were calculated according to Reed and Mencha [24,25].

### Statistical Analysis

Statistical data processing was performed using Graph Pad Prizm software version 8.0 (Graph Pad Software Inc., La Jolla, CA, USA). To evaluate the diagnostic effectiveness of the test system, ROC (Receiver Operating Characteristic) curves were analysed, with the area under the curve (AUC) determined and 95% confidence intervals calculated. Optimal cut-off values were determined using the Youden index.

Quantitative indicators were compared between several groups using analysis of variance (ANOVA), followed by Dunnett’s T3 post hoc analysis. The quality of the analysis was assessed by the coefficient of variation (CV) and the Z’-factor parameter [26,27]. The exact binomial Clopper-Pearson method was used to estimate the 95% confidence interval (CI) of specificity and sensitivity.

## 3. Results

### 3.1. Construction, Expression, and Purification of Chimeric Protein

Previously, several laboratories were engaged in mapping the structural proteins of coronaviruses to identify linear B-cell epitopes that are readily accessible for interaction with antibodies [28,29,30,31,32]. In this regard, our work identified a number of immunodominant linear B-cell epitopes, including those recognised by virus-neutralising antibodies.

**Figure 1:**
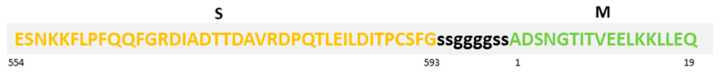
Amino acid sequence of the chimeric polyepitope peptide. The construct consists of a S protein fragment (orange), a flexible SSGGGGSS linker (black), and an M protein fragment (green), with amino acid positions shown below the sequence.

To construct a chimeric polyepitope peptide, fragments of the spike (554–593 aa) and membrane (M) (1–19 aa) proteins were used, which were connected by the SSGGGGSS sequence (Figure 1).

**Figure 2:**
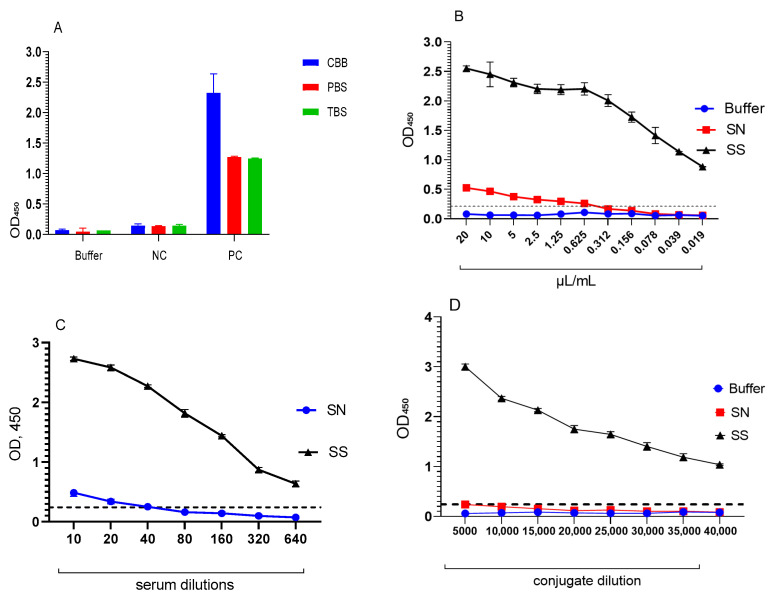
Selection of buffer solutions for sensitisation of recombinant protein (**A**); Antigenic activity of recombinant SM protein in ELISA with buffer solution for conjugate and control sera (**B**); Selection of serum dilution (**C**); Selection of conjugate dilution (**D**). CBB—carbonate-bicarbonate buffer, PBS—phosphate-buffered saline, TBS—Tris-buffered saline, NC—negative control, PC—positive control, SN—negative serum, SS—specific serum.

The synthesised nucleotide sequence encoding the chimeric peptide was cloned into the pET32b(+) expression vector. This resulted in the recombinant plasmid pET-CoV2-SM, which was transformed into *E. coli* ER2566 cells [14]. Analysis of bacterial cell lysate polypeptides showed the presence of a chimeric protein after induction of expression, whose molecular weight corresponded to the calculated value of 28 kDa. The specificity of the chimeric protein was confirmed by Western blotting using Anti-SARS-CoV-2 Spike Glycoprotein Antibody (ab272504 Abcam, Cambridge, UK) (Figure 2A; Supplementary Figure S1). The results showed that the SM protein yield was highest when using a 1:100 dilution of the overnight culture, 0.5 mM IPTG induction, and an optimal incubation time of 4 h before induction and 4 h after induction at 37 °C.

The protein was purified by metal chelate chromatography under native conditions, resulting in a purified recombinant chimeric protein preparation with a concentration of 365 μg/mL, suitable for further research on the development of an ELISA diagnostic test system (Figure 2B; Supplementary Figure S1).

### 3.2. Optimisation of the ELISA Test System Using a Chimeric Polyepitope Protein

Chimeric polyepitope protein is a key component in coating the wells of the plate in the test system for detecting antibodies to the SARS-CoV-2 virus. In this regard, we optimised the signal-to-background ratio between the positive and negative controls for all components of the assay: selection of buffer solutions for coating the plate wells (Figure 2A), determination of the working concentration of the recombinant protein (Figure 2B), and optimisation of serum dilutions (Figure 2C) and secondary antibodies (Figure 2D).

Comparison of three buffer systems: carbonate-bicarbonate buffer (CBB), phosphate-buffered saline (PBS) and tris-buffered solution (TBS) for coating of wells showed that the level of antigen binding is significantly influenced by both positive and negative serum (*p* < 0.0001) and the choice of buffer for coating (*p* = 0.0003). Among all the buffers tested, CBB showed the best result, with the best antibody binding in positive samples with high OD and low non-specific binding in negative samples. This makes it the most suitable buffer for protein dilution, and it was selected as the optimal coating buffer for the subsequent optimisation steps.

The results of the post hoc analysis using Dunnett’s T3 test showed that protein concentrations ranging from 10 to 0.312 μg/mL did not differ statistically from the maximum (20 μg/mL, *p* ≥ 0.05). Starting from 0.156 μg/mL, a significant decrease in signal was observed (*p* < 0.05). At the same time, it was noted that when using high protein concentrations in a negative sample, an increase in OD is observed, which can lead to false positive results. Therefore, the use of a minimum protein concentration of 0.312 μg/mL not only maintains reliable detection of positive samples, but also reduces non-specific binding in negative sera, increasing the specificity of the ELISA.

When diluted from 1:20 to 1:640, the OD values of negative serum exceeded the established cut-off value (cut-off = 0.213), indicating a risk of false positive results. At the same time, at a dilution of 1:100, the negative sample remained below the cut-off, and the positive sample at this dilution maintained a signal level well above the threshold and showed acceptable reproducibility. Considering the ratio of sensitivity and specificity, it was the 1:100 dilution that was determined as the working dilution for the ELISA test system.

To select the working dilution of the conjugate, eight dilution series were tested. The evaluation was based on the following criteria: a significant excess of the specific serum signal over the negative serum, the signal-to-background ratio (S/B), the ΔOD, the coefficient of variation (CV %), and the Z’-factor. The results of the analysis showed that at a dilution of 1:15,000, the specific serum signal differed significantly from the negative controls (*p* < 0.05), with a signal-to-background ratio of S/B = 13.7, a difference ΔOD = 1.975, CV = 1.5%, and Z’-factor = 0.93 reached a value of ≥0.5, which indicates high reproducibility and analytical suitability of the conjugate. This dilution was selected as the working dilution of the conjugate. The experiments showed that these conditions provide the maximum ratio between positive and negative samples when performing standard ELISA titration. Based on the data obtained, these parameters will be used in the future when setting up ELISA.

#### 3.2.1. Assessment of the Specificity of the ELISA Test System

**Figure 3:**
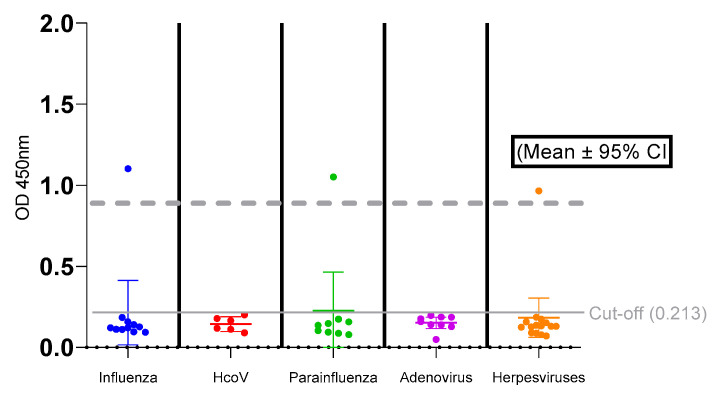
Assessment of the specificity of the ELISA test system, distribution of the reactivity index (RI) when performing ELISA based on the chimeric SM protein for SARS-CoV-2 antibody detection, results are presented for samples from patients with various viral infections (*n* = 46), the dotted line indicates the RI ≥ 0.883 threshold used to interpret a positive result.

The specificity of the test system based on recombinant SM protein for SARS-CoV-2 antibody detection was evaluated using sera containing antibodies to closely related respiratory infection pathogens, such as influenza (*n* = 11), seasonal HCoV (*n* = 6), parainfluenza (*n* = 9), adenovirus (*n* = 9), and herpesviruses (*n* = 15), as shown in Figure 3.

The results of the analysis showed that for most of the serum groups studied, the OD values were predominantly below the diagnostic cut-off (0.213). The exceptions are individual sera: one sample in the influenza, adenovirus and herpesvirus serum groups, which showed false positive results. The overall specificity of the ELISA test system was 93.5% (95% CI: 82.1–98.6%). The main characteristics of the samples are summarized in Table 2.

#### 3.2.2. Determination of Sensitivity and Specificity of the ELISA Test System

To determine the threshold value of the developed ELISA test system, ROC analysis was performed based on data from 63 negative and 63 positive serum samples obtained from humans. The constructed ROC curve demonstrated high diagnostic accuracy of the test: AUC = 0.9480 (SE = 0.01967; 95% CI: 0.9094–0.9865; *p* < 0.0001).

**Figure 4:**
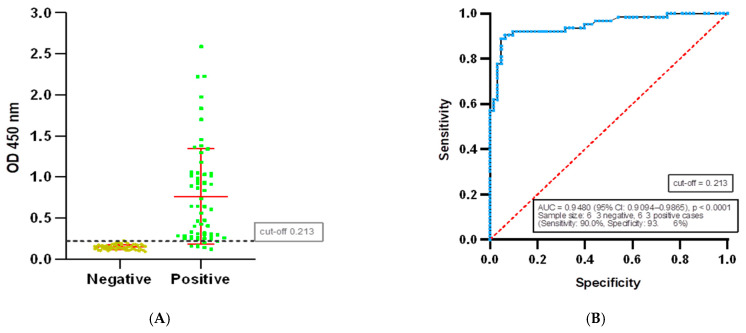
ROC analysis of the developed ELISA test system. Separation of positive and negative samples by threshold value (**A**). The ROC curve is based on 63 negative and 63 positive samples. The blue line on the graph reflects the area under the ROC curve (AUC) (**B**).

In the figure (Figure 4A), the line corresponding to the threshold value of 0.213 serves as a cut-off threshold between positive and negative results. Samples with an OD value above 0.213 are considered positive, while samples with a result below OD 0.213 are considered negative.

At a threshold of OD450 = 0.213, the sensitivity of the ELISA test system was 90.48% (95% CI: 80.74–95.56%), and the specificity was 93.65% (95% CI: 84.78–97.50%). The positive likelihood ratio (LR^+^) was 14.25, which corresponds to high diagnostic certainty of a positive result, while the integral accuracy of the test according to ROC remains high (AUC = 0.9623).

## 4. Discussion

Chimeric proteins, obtained by fusing different antigenic regions of the virus, make it possible to improve the accuracy of the diagnostic test. By incorporating multiple domains into their structure, chimeric proteins are able to bind a wide range of antibodies, which helps to increase the sensitivity of the test. On the other hand, recombinant technologies allow for the production of highly stable proteins and ensure the reproducibility of test results. The selection of recombinant proteins is important for obtaining a reliable serological test. In most serological tests for antibodies to the SARS-CoV-2 virus, the S and N proteins of this virus are used, which play a key role in viral entry and replication [6,7,8]. It is also known that the membrane protein (M) is one of the most conservative structural components of the coronavirus and plays an important role in virion morphogenesis and interaction with the immune system. Thus, in their study, Herrscher et al. [12] showed that the M protein peptide demonstrated a greater difference in reactivity between serum samples from COVID-19 patients and samples from the pre-COVID-19 period (2018). In addition, reactivity to the M protein peptide differed between groups with mild, moderate, and severe forms of COVID-19 [33]. In another study using a chimeric protein comprising SARS-CoV-2 S and N proteins, higher specificity was demonstrated, reaching 100% in the detection of antibodies in serum and urine. However, sensitivity was 83.7% when using serum samples and 91.1% when using urine samples [9]. Given the constant emergence of new variants of the SARS-CoV-2 virus, the development of a diagnostic test capable of detecting a wide range of antibodies is a pressing task.

To assess the stability and conservation of the selected epitopes, they were aligned with the reference sequences of the main SARS-CoV-2 variants (Wuhan-Hu-1, Alpha, Beta, Delta, Omicron). The M-protein epitope proved to be highly conservative is conserved across the analysed variants (Figure 3, Supplementary Figure S1). The S-protein epitopes also demonstrate a high degree of conservation: their sequences coincide in most variants, confirming the stability of these protein regions (Figure 2, Supplementary Figure S1). These data underscore the robustness and suitability of the selected epitopes for use in serological tests and immune response studies.

In this study, we used a recombinant chimeric SM protein we designed for the SARS-CoV-2 as an antigen for developing an ELISA to detect specific antibodies in human blood serum. The chimeric antigen, which includes linear epitopes from the S and M proteins, was chosen for its complementary advantages. The S-protein region in our construct is located in a relatively conservative and less mutation-prone segment, which may ensure stable detection of antibodies to different variants of SARS-CoV-2. The inclusion of the M-protein allows detection of additional linear antibody responses, expanding serological coverage. In addition, the use of only recombinant S protein in serological tests may result in reduced sensitivity, especially in populations with atypical clinical presentations or delayed seroconversion. The S protein was chosen because it has high immunogenicity, as demonstrated by numerous studies [9,15,31,32]. The use of SARS-CoV-2 M protein epitopes as a diagnostic test is rare. However, studies show that the level of antibody response recognising linear epitopes of the M protein is in the same range as for the S and N epitopes of the SARS-CoV-2 virus protein [14]. Previous work has been done on mapping linear B-cell epitopes of coronavirus infection proteins. Using peptide microarrays, epitopes of the spike (S) and membrane (M) proteins of SARS-CoV-2, recognised by the sera of a large number of recovered patients, were characterised. Analysis showed that individual linear epitopes of the S protein, as well as immunodominant epitopes of the M protein, were recognised by antibodies in patient sera, i.e., antibodies capable of blocking the infectivity of the virus. These data allow us to identify functionally significant protein regions that can be used to develop peptide vaccines and diagnostic tests aimed at detecting neutralising antibodies [28]. Our results confirmed that this chimeric protein containing S and M protein epitopes has high antigenic activity and allows reliable differentiation between positive and negative samples.

In accordance with this approach, after selecting a chimeric protein combining the immunodominant regions of the S and M proteins, we optimised the parameters of the developed ELISA test system. This allowed us to improve the OD signal ratio and increase the specificity of the test without reducing its sensitivity. The obtained parameters were confirmed by ROC analysis data, according to which the integral accuracy of the test system remained high (AUC = 0.9623), and the positive likelihood ratio (LR+) significantly exceeded the diagnostically significant threshold values.

With an optimised cut-off value (OD_450_ = 0.213), our test system achieved a specificity of ~93.65% (95% CI: 84.78–97.50%) in initial evaluations (Figure 3). From a practical point of view, such high specificity is crucial for preventing false positive results, which is an important property of a confirmatory serological test. For comparison, many early commercial tests for antibodies to the SARS-CoV-2 virus demonstrated a specificity of ~95%, which could still lead to some false positive results [34]. Thus, increasing the specificity to 98–99% represents a significant improvement in the reliability of the ELISA test system.

The results of the specificity of the ELISA test system we developed (Figure 4) with an overall specificity of ~93.5% is consistent with previously known literature data on highly specific ELISA test systems for the SARS-CoV-2 virus (specificity ≈99% and minimal cross-reactivity to HIV, HBV, CMV, EBV, and other panels) [34]. Isolated false-positive reactions are likely due to partially overlapping immune reactivity of epitopes and the presence of cross-reactive antibodies described in pre-pandemic sera (seasonal HCoV) [34]. In addition, false positive results in serological tests for SARS-CoV-2 may be caused by interference from autoantibodies (rheumatoid factor, ANA) in patients with chronic inflammatory diseases [12,34,35,36,37,38,39].

Therefore, the high sensitivity of our test system indicates that a positive result is a highly reliable confirmation of the presence of antibodies specific to the SARS-CoV-2 virus, and the probability of false detection of antibodies to unrelated concomitant infections is very low.

The results of the study of 126 samples (Figure 4) confirm the diagnostic efficacy of the serological test for antibodies to the chimeric SM SARS-CoV-2 protein at a threshold of OD450 = 0.213. The sensitivity obtained (90.48%, 95% CI: 80.74–95.56%) indicates the ability of the test system to detect true positive cases, which is extremely important for determining the immune status of patients after infection or vaccination.

The specificity of 93.65% (95% CI: 84.78–97.50%) minimises the number of false positive results, ensuring accurate interpretation and reducing the risk of misjudging immunity. A positive diagnostic ratio (LR+) of 14.25 indicates that a positive test result with a high score corresponds to the presence of antibodies. A value significantly higher than the established threshold (>10) indicates the high effectiveness of the test system in detecting a specific immune response to this pathogen.

The area under the ROC curve (AUC = 0.9623) confirms the high integral accuracy of the test system and its ability to effectively separate positive and negative samples, ensuring stability and reproducibility of results in different populations and study conditions.

The studies conducted have some limitations, such as the evaluation of the test for the diagnosis of active infection and long-term immunity, which we plan to conduct in the future. This would provide a more complete picture of the clinical capabilities of the developed test system and allow us to assess its applicability in broader conditions.

Consequently, our data confirm the feasibility of using the chimeric SM protein in the ELISA test system for serological testing, which opens up prospects for further research and scaling of this approach. Thanks to the sensitivity and specificity indicators presented above, the ELISA test system we have developed can be effectively used to assess the specific immune response to the SARS-CoV-2 virus and to conduct both laboratory and monitoring studies.

## 5. Conclusions

During the study the ELISA test system based on a chimeric polyepitope peptide (SM) was developed, using fragments of spike and matrix proteins that were linked together by the S2G4S2 sequence. The optimisation stage of the ELISA test system included the selection of antigen concentration, conditions, incubation and serum dilution, which allowed us to achieve high sensitivity and specificity of the test system. The test system demonstrated high sensitivity of 90.48% and specificity of 93.65%, confirming its reliability in detecting antibodies for SARS-CoV-2. The developed method has prospects for wide application in clinical diagnostics and monitoring of the immune status of the population.

## Figures and Tables

**Table 1:** Serum samples used to determine the cross-reactivity of the ELISA test system.

№	Sample Category	Etiological Agent/Subgroup	Number of Samples	Storage Temperature
1	Seasonal sera, pre-pandemic	HcoV	6	−80 °C
2	Cross-reactive (respiratory viruses)	Influenza	11	−80 °C
		Parainfluenza	9	−80 °C
		Adenovirus	9	−80 °C
3	Cross-reactive (other)	Herpesviruses	15	−80 °C

**Table 2:** Specificity of the ELISA test system.

Sera/Infection	Number of Samples, *n*	False-Positive Samples (FP)	Specificity, % (95% CI)
Influenza	11	1	90.9 (58.7–99.8)
Seasonal HCoV, pre-pandemic	5	0	100 (47.8–100)
Parainfluenza	6	0	100 (54.1–100)
Adenovirus	9	1	88.9 (51.8–99.7)
Herpesviruses	15	1	93.3 (68.0–99.8)
Total	46	3	93.5 (82.1–98.6)

## Data Availability

The data presented in this study are available from the corresponding author upon reasonable request.

## Supplementary Materials

The following supporting information can be downloaded at: https://www.mdpi.com/article/10.3390/mps9010004/s1, Figure S1: Immunodetection of *E. coli* cell lysate proteins (A). M—molecular weight protein marker; 1,2—cell lysates before induction; 3,4,5—cell lysates after expression induction. Electrophoretic analysis of purified recombinant SM protein (B). M—molecular weight protein marker; 1—purified SM protein.

## Abbreviations

The following abbreviations are used in this manuscript:

SARS-CoV-2	Severe Acute Respiratory Syndrome Coronavirus 2
COVID-19	Coronavirus Disease 2019
ELISA	Enzyme-Linked immunosorbent assay
PCR	Polymerase Chain Reaction
BSA	Bovine serum albumin
TMB	3,3’,5,5’-tetramethylbenzidine
IPTG	Isopropyl β-D-1-thiogalactopyranoside
CBB	Carbonate-bicarbonate buffer
PBS	Phosphate-buffered saline
TBS	Tris-buffered solution
OD	Optical density
VNT	Virus neutralisation test
