# Supplementary material for: Development of an ELISA Using Recombinant Chimeric SM Protein for Serological Detection of SARS-CoV-2 Antibodies
Source: Methods Protoc. 2025 Dec 22;9(1):4. doi: 10.3390/mps9010004 (PMC12821618; doi:10.3390/mps9010004)
Supplement: Supplementary file 1 [file mps-09-00004-s001.zip › mps-3939244-supplementary.pdf]

A B

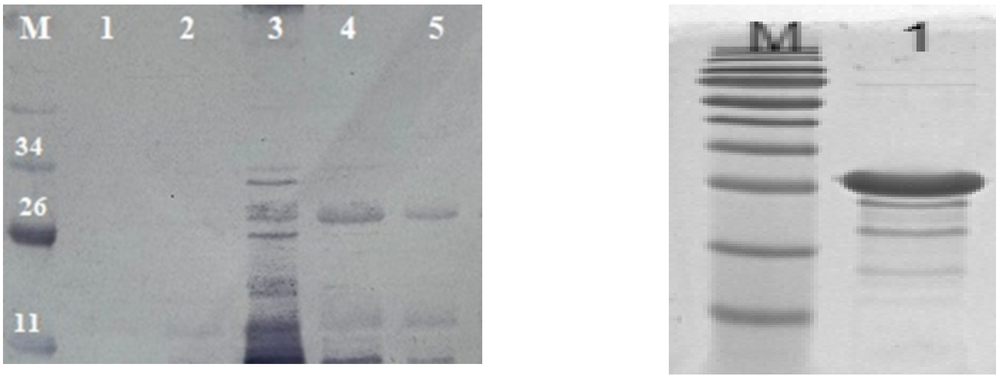

Figure S1. Immunodetection of E. coli cell lysate proteins (**A**). M – molecular weight protein marker; 1,2 – cell lysates before induction; 3,4,5 – cell lysates after expression induction. Electrophoretic analysis of purified recombinant SM protein (**B**). M – molecular weight protein marker; 1 – purified SM protein.
